# Exploring local perceptions and drivers of engagement in biodiversity monitoring among participants in payments for ecosystem services schemes in southeastern Mexico

**DOI:** 10.1111/cobi.14282

**Published:** 2024-04-25

**Authors:** Santiago Izquierdo‐Tort, Andrea Alatorre, Paulina Arroyo‐Gerala, Elizabeth Shapiro‐Garza, Julia Naime, Jérôme Dupras

**Affiliations:** ^1^ Instituto de Investigaciones Económicas Universidad Nacional Autónoma de México Mexico City Mexico; ^2^ Département Des Sciences Naturelles Université du Québec en Outaouais Ripon Quebec Canada; ^3^ Institute of Development Policy University of Antwerp Antwerpen Belgium; ^4^ Natura y Ecosistemas Mexicanos AC Mexico City Mexico; ^5^ Nicholas School of the Environment Duke University Durham North Carolina USA; ^6^ Center for International Forestry Research Bogor Indonesia; ^7^ Institut des Sciences de la Forêt tempérée, Université du Québec en Outaouais Université du Québec en Outaouais Ripon Quebec Canada

**Keywords:** biodiversity conservation, Chiapas, conservation payments, Mexico, participatory environmental monitoring, payments for ecosystem services, stakeholder engagement, pagos por servicios ambientales, conservación de la biodiversidad, monitoreo ambiental participativo, participación de las partes interesadas, pagos por conservación, Chiapas, México

## Abstract

Payments for ecosystem services (PES) are widely applied incentive‐based instruments with diverse objectives that increasingly include biodiversity conservation. Yet, there is a gap in understanding of how to best assess and monitor programs’ biodiversity outcomes. We examined perceptions and drivers of engagement related to biodiversity monitoring through surveys among current PES participants in 7 communities in Mexico's Selva Lacandona. We conducted workshops among survey participants that included training and field deployment of tools used to monitor biodiversity and land cover, including visual transects, camera traps, acoustic recorders, and forest cover satellite images. We conducted pre‐ and postworkshop surveys in each community to evaluate changes in respondents’ perceptions following exposure to biodiversity monitoring training and related field activities. We also reviewed existing research on participatory environmental management and monitoring approaches. One quarter of current PES participants in the study area participated in our surveys and workshops. The majority stated interest in engaging in diverse activities related to the procedural aspects of biodiversity monitoring (e.g., planning, field data collection, results dissemination) and acknowledged multiple benefits of introducing biodiversity monitoring into PES (e.g., knowledge and capacity building, improved natural resource management, and greater support for conservation). Household economic reliance on PES was positively associated with willingness to engage in monitoring. Technical expertise, time, and monetary constraints were deterrents. Respondents were most interested in monitoring mammals, birds, and plants and using visual transects, camera traps, and forest cover satellite images. Exposure to monitoring enhanced subsequent interest in monitoring by providing respondents with new insights from their communities related to deforestation and species’ abundance and diversity. Respondents identified key strengths and weaknesses of applying different monitoring tools, which suggests that deploying multiple tools simultaneously can increase local engagement and produce complementary findings and data. Overall, our findings support the relevance and usefulness of incorporating participatory biodiversity monitoring into PES.

## Abstract (es)

Explorando las percepciones locales y los impulsores de la participación en el monitoreo de la biodiversidad entre participantes de esquemas de pagos por servicios ambientales en el sureste de México

**Resumen:**

Los pagos por servicios ambientales (PSA) son instrumentos basados en incentivos ampliamente aplicados cuyos diversos objetivos incluyen cada vez más la conservación de la biodiversidad. Sin embargo, existe una brecha en la comprensión de cómo evaluar y monitorear los resultados de los programas en materia de biodiversidad. Examinamos las percepciones y los impulsores de la participación relacionados al monitoreo de la biodiversidad a través de encuestas entre participantes actuales de PSA en siete comunidades de la Selva Lacandona de México. Realizamos talleres entre los participantes de las encuestas que incluyeron capacitación y despliegue en campo de herramientas utilizadas para monitorear la biodiversidad y la cobertura del suelo, incluidos transectos visuales, cámaras trampa, grabadores acústicos e imágenes satelitales de la cobertura forestal. Realizamos encuestas antes y después del taller en cada comunidad para evaluar los cambios en las percepciones de los participantes tras su involucramiento en la capacitación de monitoreo de biodiversidad y las actividades de campo relacionadas. También revisamos la investigación existente sobre enfoques de monitoreo y gestión ambiental participativa. Una cuarta parte de los participantes actuales de PES en el área de estudio participaron en nuestras encuestas y talleres. La mayoría declaró interés en participar en diversas actividades relacionadas con los aspectos procesales del monitoreo de la biodiversidad (por ejemplo, planificación, recopilación de datos de campo, difusión de resultados) y reconoció múltiples beneficios de introducir el monitoreo de la biodiversidad en los PSA (por ejemplo, creación de conocimientos y capacidades, mejora de la gestión de los recursos naturales, y mayor apoyo a la conservación). La dependencia económica de los hogares de los PSA se asoció positivamente con la voluntad de participar en el monitoreo. Los factores inhibidores fueron la experiencia técnica, el tiempo y las limitaciones monetarias. Los encuestados estaban más interesados en monitorear mamíferos, aves y plantas y en utilizar transectos visuales, cámaras trampa e imágenes satelitales de la cubierta forestal. La exposición al monitoreo aumentó el interés posterior en el monitoreo al brindar a los encuestados nuevos conocimientos de sus comunidades relacionados con la deforestación y la abundancia y diversidad de especies. Los encuestados identificaron fortalezas y debilidades clave al aplicar diferentes herramientas de monitoreo, lo que sugiere que implementar múltiples herramientas simultáneamente puede aumentar la participación local y producir hallazgos y datos complementarios. En general, nuestros hallazgos respaldan la relevancia y utilidad de incorporar el monitoreo participativo de la biodiversidad en los PSA.

## INTRODUCTION

Payments for ecosystem services (PES) are incentive‐based instruments that provide payments conditional on specific natural resource management activities, such as forest conservation (Kolinjivadi et al., 2023; Salzman et al., 2018). They are being increasingly integrated into existing conservation efforts to finance biodiversity conservation and provide other ecosystem services, such as water provision and carbon capture (Rudolf et al., 2022; Wunder & Wertz‐Kanounnikoff, 2009; Wunder et al., 2018; Xu et al., 2021). Yet, the role of biodiversity conservation within the ecosystem services framework has been contested in conceptual and practical terms (Lele et al., 2013). Biodiversity conservation is a stated goal in a growing number of PES programs (Ezzine‐de‐Blas et al., 2016; Salzman et al., 2018), but, apart from a few exceptions, there has been little tracking of PES outcomes related to biodiversity (Bremer et al., 2019; Chen et al., 2020).

Expected contributions of PES to biodiversity conservation depend on required activities and the definition of *biodiversity* adopted (Hein et al., 2013). Most large‐scale PES programs in biodiverse countries, such as Mexico, Costa Rica, and Ecuador, focus on maintaining or restoring native ecosystems and habitats, often forests, by offering conditional payments to landowners and communities to avoid land‐use changes or engage in restoration (Jones et al., 2020; Shapiro‐Garza, 2020). This approach intends to safeguard biological diversity on PES lands, without further specification (Bremer et al., 2019), and to provide cobenefits in the form of ecosystem services (Wunder & Wertz‐Kanounnikoff, 2009). However, evaluating PES contributions to biodiversity in this approach is complicated because specific biodiversity goals and metrics of success are rarely stated (Bremer et al., 2019). More direct, though less frequent, approaches also exist whereby programs provide payments for protecting or managing specific species or taxonomic groups (Chakrabarti et al., 2019; Clements et al., 2010).

Contributions of PES to biodiversity conservation thus remain largely unknown (Baylis et al., 2016; Börner et al., 2017). Programmatic and scholarly performance evaluations of PES schemes have relied on offsite data (e.g., remote sensing) and indirect proxies (e.g., forest cover) rather than direct indicators of biodiversity or other ecosystem services (Kaiser et al., 2021; Naeem et al., 2015; Prager et al., 2016). This reflects broader deficiencies in monitoring biodiversity outcomes in conservation efforts, particularly in highly biodiverse regions (Hochkirch et al., 2021; Mammola et al., 2023; Schmeller et al., 2017; Stephenson, 2020).

We examined local perceptions and drivers of engagement in biodiversity monitoring among current participants in PES schemes in a case study in Mexico. Because PES schemes are often applied in heavily managed or inhabited areas (Kerr et al., 2014; Robinson et al., 2016), employing participatory monitoring approaches can enhance conservation awareness and policy buy‐in among local PES participants (Rakotomahazo et al., 2019; Schröter et al., 2018). Also, adopting participatory monitoring approaches can help understanding of the concept, values, and indicators associated with biodiversity conservation as a PES goal for different stakeholders (Lele et al., 2013). We took advantage of technological advancements and improved access to and availability of tools for biodiversity monitoring (Hochkirch et al., 2021; Nuñez et al., 2019; Stephenson, 2020). We also drew from the literature that emphasizes the instrumental and intrinsic benefits of participatory monitoring approaches (Andrade & Rhodes, 2012; Becker et al., 2005; Danielsen et al., 2010; Fernandez‐Gimenez et al., 2008; Rakotomahazo et al., 2019; Sterling et al., 2017) and the factors that influence local willingness to engage in environmental management and monitoring (Brites & Morsello, 2018).

Our approach combined analyses of existing research on participatory environmental management and monitoring approaches and data collection and field activities among PES participants in 7 communities in Mexico. Activities included workshops that provided training and field deployment of tools used to monitor biodiversity and land cover and pre‐ and postworkshop surveys of people's monitoring interests, previous experiences, and perceptions. We asked 3 research questions: What are the relative advantages and engagement requirements for adopting participatory biodiversity monitoring approaches in PES? Which biodiversity monitoring tools are relevant for PES? How do current PES participants perceive and engage with biodiversity monitoring?

## ADOPTING PARTICIPATORY BIODIVERSITY MONITORING APPROACHES IN PES

### Benefits and pitfalls of local engagement

The scope for promoting local engagement is significant because most PES programs are developed as top‐down instruments by policy makers and scientists (Salzman et al., 2018), despite growing calls for local participation in environmental policy‐making processes and the production of contextually situated knowledge (Bennett, 2016; Wyborn & Evans, 2021).

Applying participatory biodiversity monitoring approaches in PES can help produce locally specific and high‐quality biodiversity data that can be used to determine program conservation status and support the implementation of more contextually relevant conservation activities (Danielsen et al., 2021; Krause & Zambonino, 2013). These data can be used reliably to document the conservation status of biodiversity in a PES setting and help identify key improvements for policy design and implementation (Becker et al., 2005; Beirne et al., 2019; Theobald et al., 2015), such as revealing specific areas where environmental protection is crucial due to the presence of certain species or defaunation processes.

Another potential benefit relates to the scope of participatory monitoring approaches for enhancing PES performance and changing participant attitudes and actions toward conservation. Beyond simply collecting data, local populations can participate in other decision‐making processes and activities, including planning and scoping, activity design and implementation, data analyses and management, and result dissemination (Shirk et al., 2012). Local engagement in activities and decision‐making processes can enhance policy benefits by inducing higher compliance (Andrade & Rhodes, 2012), increasing speed of implementation (Danielsen et al., 2010), improving collective buy‐in and ownership (Rakotomahazo et al., 2019; Schröter et al., 2018), improving attitudes toward conservation (Sterling et al., 2017), lowering risk of crowding out of intrinsic conservation motivations (Upton, 2020), and improving the quality of environmental decisions (Reed, 2008). From the local populations’ perspective, additional benefits of engaging in decision‐making processes include knowledge and information (Fernandez‐Gimenez et al., 2008; Krause & Zambonino, 2013; Newman et al., 2003), community empowerment and sovereignty (Danielsen et al., 2021), leadership and social capital (Becker et al., 2005), and a higher probability that processes are perceived as locally fair and legitimate (Cavalcanti et al., 2010; Wilson et al., 2018).

Finally, adopting biodiversity monitoring approaches in PES could help build support for program implementation. In recent years, some PES programs have experienced defunding and budget cuts (Etchart et al., 2020; Hayes et al., 2022; Rode, 2022). By providing additional data and insights on biodiversity outcomes and other on‐the‐ground ecological dynamics, biodiversity monitoring approaches can help build support for PES among funders and the public.

However, there are potential pitfalls to introducing monitoring in local contexts that are relevant for PES: accidentally or deliberately collecting data on human subjects without their consent (Sandbrook et al., 2021; Sharma et al., 2020); compromising the physical or psychological safety of collectors, where field activities cause accidents or retaliation by poachers or other actors whose illegal activities become exposed (Tomaszewski & Kołakowski, 2023); reproducing or exacerbating inequalities, where benefits associated with monitoring (e.g., access to decision‐making processes, training and capacity building, equipment or other economic resources) accrue disproportionately among local elites (Clements et al., 2010; Izquierdo‐Tort et al., 2022; Sommerville et al., 2010; Staddon et al., 2015); and creating dependency on external support (i.e., local populations become reliant on external actors in terms of tools, technical support, or other resources) (Pritchard, 2013).

### Local willingness to engage

The adoption and success of participatory biodiversity monitoring approaches depend on individual‐ and community‐level attributes that influence willingness to engage in monitoring activities (Aswani et al., 2013; Brites & Morsello, 2018; Cavalcanti et al., 2010; Maskey et al., 2006). Although PES studies that examine the specific aspect of biodiversity monitoring are scarce (Bremer et al., 2019; Chen et al., 2020), the idea of incorporating biodiversity monitoring entails some issues that have been relatively well‐documented in PES literature, including drivers of program enrollment (Jones et al., 2020), influence of monetary and nonmonetary incentives on participant behavior (Akers & Yasué, 2019), and equity aspects of community engagement (Loft et al., 2020).

Generally, individuals voluntarily engage in monitoring and management activities if perceived benefits outweigh costs. Actual or expected economic benefits or costs can be important drivers of individual engagement in environmental monitoring (Evans & Guariguata, 2008; Maskey et al., 2006) and PES programs (Authelet et al., 2021; Naime et al., 2024). Particularly, those who benefit or depend more on natural resources have a greater disposition to participate in monitoring and management activities (Brites & Morsello, 2018; Dalton et al., 2012). A broader set of noneconomic and prosocial motivations (e.g., autonomy, learning opportunities, social norms, cooperative behavior) can either enhance or hinder engagement in monitoring and management activities (Aswani et al., 2013; Brites & Morsello, 2018) or participation in PES programs (Akers & Yasué, 2019; Authelet et al., 2021). Although the importance of community participation in PES to address equity concerns is recognized (Pascual et al., 2014), there is mixed evidence on the effectiveness of community approaches in monitoring and enforcement in the frame of collective schemes (Eisenbarth et al., 2021; Kaczan et al., 2017; Naime et al., 2022), the role of nonmonetary incentives and potential for program enrolment to crowd out intrinsic motivations (Diendéré & Kaboré, 2023; Ezzine‐de‐Blas et al., 2019; Rode et al., 2015), and the risk of benefits associated with program enrollment, such as economic payments or training in natural resources management, being disproportionately captured by a few participants (Izquierdo‐Tort et al., 2022; Milne & Adams, 2012).

Motivations aside, a key issue is whether activities can attract and sustain sufficient local interest for successful task completion (Brites & Morsello, 2018). Although some monitoring activities, such as field data collection, can be achieved in the short term with a small group of collectors, longer term success requires ensuring continued support or replacement of volunteers (Brites & Morsello, 2018). Key factors that can threaten long‐term involvement in environmental monitoring include lack of economic incentives and technical skills, fatigue, boredom, and risks to the physical or psychological safety of collectors (Gabillet et al., 2020; Soller et al., 2020; Tomaszewski & Kołakowski, 2023). Involving local individuals or groups in decision‐making processes is an important factor that can help sustain engagement in environmental monitoring (Cavalcanti et al., 2010) and enrollment and outcomes in PES schemes (Bremer et al., 2023; Lliso et al., 2022).

Although local monitors in a PES context can include households with enrolled lands or other inhabitants, we prioritized enrolled landholders because local people are likely to possess the best insights on enrolled lands; participation is self‐selected and thus indicates some interest in biodiversity conservation; and enrollment information can be accessed, thus enabling fieldwork and analyses. We recognize, however, that such selection has potential drawbacks for data reliability which we considered (see “DISCUSSION”).

## RELEVANT TOOLS FOR PARTICIPATORY BIODIVERSITY MONITORING IN PES

We identified potentially useful and suitable tools for participatory biodiversity monitoring in a PES context based on a review of existing literature and subsequent ranking of the tools identified according to the reviewed literature's assessments and our own experience.

### Relevant tools and evaluation criteria

Biodiversity monitoring encompasses different tools for collecting and analyzing information related to the spatial, temporal, and taxonomic coverage of biodiversity. Tools are of 3 main types: traditional observer‐based (relies on direct human observation and includes methods such as specimen collection and visual transects); remote sensing (involves satellite or aerial imagery to study phenomena such as forest cover change or 3‐dimensional analyses of forest structure [e.g., laser imaging detection and ranging, lidar]); and earth‐based sensors (collect data autonomously and include camera traps [Green et al., 2020], drones, acoustic recorders, and environmental DNA [e‐DNA] [Bohmann et al., 2014]). Recent technological breakthroughs offer opportunities for enhanced data collection and analyses in highly biodiverse but data‐deficient regions, such as tropical forests (Hochkirch et al., 2021; Schmeller et al., 2017; Stephenson, 2020). Further, increased access to and reduced cost of a wider range of satellite‐based remote sensing data (e.g., Planet data) and earth‐based sensors complement traditional observer‐based methods (Nuñez et al., 2019; Stephenson, 2020).

Typical criteria for selecting which tools are useful for biodiversity monitoring in a specific context include scientific and practical considerations, such as the objective of monitoring, types of users involved, taxonomic focus, and financial, technical, and legal constraints (Chandler et al., 2017; Mulatu et al., 2017; Seak et al., 2012; Sullivan & Molles, 2016). Financial and technical feasibility are particularly important in a PES context because the responsibility for purchasing equipment and financing other costs related to biodiversity monitoring (e.g., field collection and training, community engagement, data analysis and management, reporting and dissemination) would ideally not fall disproportionately on program implementers nor participants. As Mexico's PES experience shows, program implementers may struggle to secure sufficient funding over time, and imposing additional burdens on participants may create discontent and even deter enrollment (Izquierdo‐Tort et al., 2021). Cost‐sharing arrangements could be potentially built with other parties interested in biodiversity monitoring, such as research centers, nongovernmental organizations (NGOs), or international organizations (Stephenson et al., 2017), to ensure sufficient and sustained funding for each task (Collen et al., 2013).

Technical requirements for the adequate deployment of equipment include basic infrastructure and connectivity needs (e.g., physical access, electricity), as well as the level of skills needed for each tool and the ease of adoption (Schmeller et al., 2017). Ensuring these conditions are met in a PES setting would be particularly challenging for tools, such as remote sensing, that require high technical skills (e.g., software proficiency) and whose use has a high learning curve.

Biodiversity monitoring tools and approaches should additionally align with specific PES design and implementation features. Although program features may vary significantly across contexts (Engel, 2016), we identified 2 characteristics that are distinct to PES that are relevant for selecting biodiversity monitoring approaches and tools. First, if biodiversity conservation is an explicit PES goal, monitoring tools should be able to produce data that can reliably evaluate participant compliance or lack thereof with previously agreed‐upon actions or outcomes related to biodiversity. Indicators can be indirect (e.g., land cover or land use) or direct, such as the spatial or temporal distribution or abundance of certain animal or plant species (e.g., keystone species) or taxonomic groups (e.g., mammals, birds).

Second, monitoring tools should be able to produce data in a time frame consistent with the length of the PES contract and the periodicity of compliance assessments and payment distributions. Because most PES contracts are short term (e.g., 3–5 years) and provide periodic payments (e.g., annual), tools that can produce biodiversity measurements within this time period would be best suited to assess at least the programs’ short‐term performance. Because PES contracts are spatially explicit, that is, lands enrolled are demarcated to a specific geographic area, monitoring tools should be able to match the spatial coverage of the contract.

### Relative advantages of specific tools

Table 1 presents our interpretation of the relative suitability of several monitoring tools across traditional observer‐based, remote sensing, and earth‐based sensors. Given our focus on PES programs that aim to conserve native ecosystems (forests in our Mexican case study), we selected a range of tools that can capture both the general state of the ecosystem and the biological diversity therein in terms of specific species or taxonomic groups. We qualitatively ranked the usefulness or suitability of each tool (i.e., high, medium, low) according to categories based on 4 criteria: financial feasibility, technical feasibility, consistency with payment conditionality, and consistency with contract spatiotemporal coverage.

**TABLE 1:** Relative advantages of biodiversity monitoring tools for application in a payment for ecosystem services context.

			Traditional observed based		Remote sensing	Earth‐based sensors			
Category	Criterion	Subcriterion or description	Specimen collection	Visual transects	Forest cover satellite images, aerial, lidar	Camera traps	Drones	Acoustic recorders	e‐DNA
PES specific	Payment conditionality	Ability to evaluate compliance with chosen program outcome	Medium	Medium	High (4)	High	Medium	High	High
		Taxonomic groups that can be monitored	Mammals, birds, invertebrates (5,6,9)	Birds, fish, mammals, reptiles (1,3,5,6)	Plants, large animals, or animal groups (mammals or birds) (2,4)	Mammals, birds (smaller and aquatic species not easily detected (2,8)	Plants, large animals, or animal groups (mammals or birds) (2,8)	Vocal species of birds, amphibians, insects, mammals (2,8)	Plants, mammals, insects, birds, amphibians, reptiles, mollusks, fish (2,7,8)
	Contract temporality and spatial coverage	Ability to match short‐term contract timeline	High	Medium	High (4)	High	High	Medium (2)	Low
		Ability to match spatial coverage of contract	Medium	Low (2)	High (4)	High (10)	Medium	High (2,8)	Low (2)
General	Financial feasibility	Costs of equipment for data collection	Medium (1)	High (1,9)	Medium (2)	Medium (2,10)	Low (9)	Low (2,9)	Low (2,7,9)
		Costs of data analysis	Medium	Medium	Low	Low (10)	Low	Low (2)	Low (4)
	Technical feasibility	Technical skills required for data collection	High (5,9)	High (5,9)	Low (2	Medium (8)	Medium (8,9)	Medium (2,8,9)	Medium (9)
		Connectivity and infrastructure requirements	Medium	High	Low (2)	Low (8)	Low (8)	Low (8)	Low
		Technical skills required for data analysis	Low (9)	Medium	Low (2)	Medium (4,8,10)	Low (8,9)	Medium (4,8)	Low (4,7)
		Invasiveness	Low	High	High	High	High	High	High

*Note*: High, medium, and low indicate the relative usefulness or suitability of a monitoring technique or tool. For instance, low costs of data analyses indicate costs are low and therefore applying the tool is highly feasible. Numbers in parentheses correspond to citations: 1, Seak et al. (2012); 2, Stephenson (2020); 3, Clements et al. (2010); 4, Mulatu et al. (2017); 5, Newman et al. (2003); 6, Becker et al. (2005); 7, Bohmann et al. (2014); 8, Schmeller et al. (2017); 9, Sullivan and Molles (2016); 10, Green et al. (2020). No citation indicates the classification is based on the authors’ prior experience with biodiversity monitoring and PES research.

Observer‐based tools offer high financial and technical feasibility and can match the temporal timeline of PES’ contracts. Yet, a key pitfall is their limited spatial scope. In contrast, remote sensing tools are particularly useful for monitoring outcomes across different temporal and spatial scales. However, high cost and need for highly specialized training for data collection and analyses make remote sensing tools less suitable for participatory approaches. Finally, earth‐based sensors, such as camera traps and acoustic recorders, score relatively well across all PES‐specific categories due to their high taxonomic range, the fact that equipment can be placed on site and collect data uninterruptedly, and the optimal placement of equipment achieved by engaging local landowners familiarized with the local context. The cost of acquiring and deploying equipment, together with knowledge barriers for analyzing the data, makes earth‐based sensors unsuitable in contexts with low resources and technical capabilities. Through our analysis, we sought to suggest trade‐offs across traditional, remote sensing, and earth‐based tools for biodiversity monitoring in a PES context.

## METHODS

### Study area

We explored local perceptions and engagement related to biodiversity monitoring among 7 communities that participate in PES programs in Mexico. The study site was the municipality Marqués de Comillas (MdC) in Selva Lacandona, Chiapas, a tropical forest frontier and a biodiversity hotspot (Carabias et al., 2015). Landholders in MdC have participated in several national (i.e., biodiversity and hydrological) and subnational (i.e., Selva Lacandona Special Program) public PES programs since the late 2000s (Izquierdo‐Tort et al., 2021). Mexico's PES programs are managed by the National Forestry Commission (CONAFOR) and involve 5‐year contracts with annual payments per hectare. Taken together, the various types of public PES in Mexico represent some of the largest and longest standing initiatives worldwide (Shapiro‐Garza, 2020).

Participating in CONAFOR's PES scheme requires satisfying legal, technical, ecological, and socioeconomic criteria (e.g., lands having over 70% forest cover in an area with high marginalization levels) and a high ranking among other applicants based on assigned points (Izquierdo‐Tort et al., 2021). To maintain enrollment, participants must propose and implement a best management practices guide that includes mandatory (e.g., maintaining forest cover, avoiding land cover change, performing periodic surveillance, developing fire control measures) and recommended measures (e.g., water regulation and soil conservation works, control of exotic or non‐native species) (Avila‐Foucat et al., 2021; CONAFOR, 2023; Jones et al., 2018). Though CONAFOR allocates specific resources to hire technical advisors that help participants plan field activities and prepare annual activity reports (Jones et al., 2018), satisfying program requirement is costly for participants in terms of time, labor, and money (Alix‐Garcia et al., 2018; Izquierdo‐Tort et al., 2021; Rodríguez‐Robayo et al., 2016).

The PES’ program rules (e.g., CONAFOR, 2023) are not explicit as to how activities should contribute to biodiversity conservation, but they do so implicitly through habitat management and ecosystem conservation (preventing land cover or use change and implementing management practices that support wildlife, such as revegetation); community engagement and capacity building (supporting education and training activities, strengthening natural resource governance, and promoting sustainable productive practices); and ecological monitoring and construction of wildlife infrastructure (conducting surveillance tours to detect natural and human‐related risks and constructing wildlife support infrastructure, such as observation posts, feeders, and watering holes).

### PES participant surveys and workshops

**FIGURE 1:**
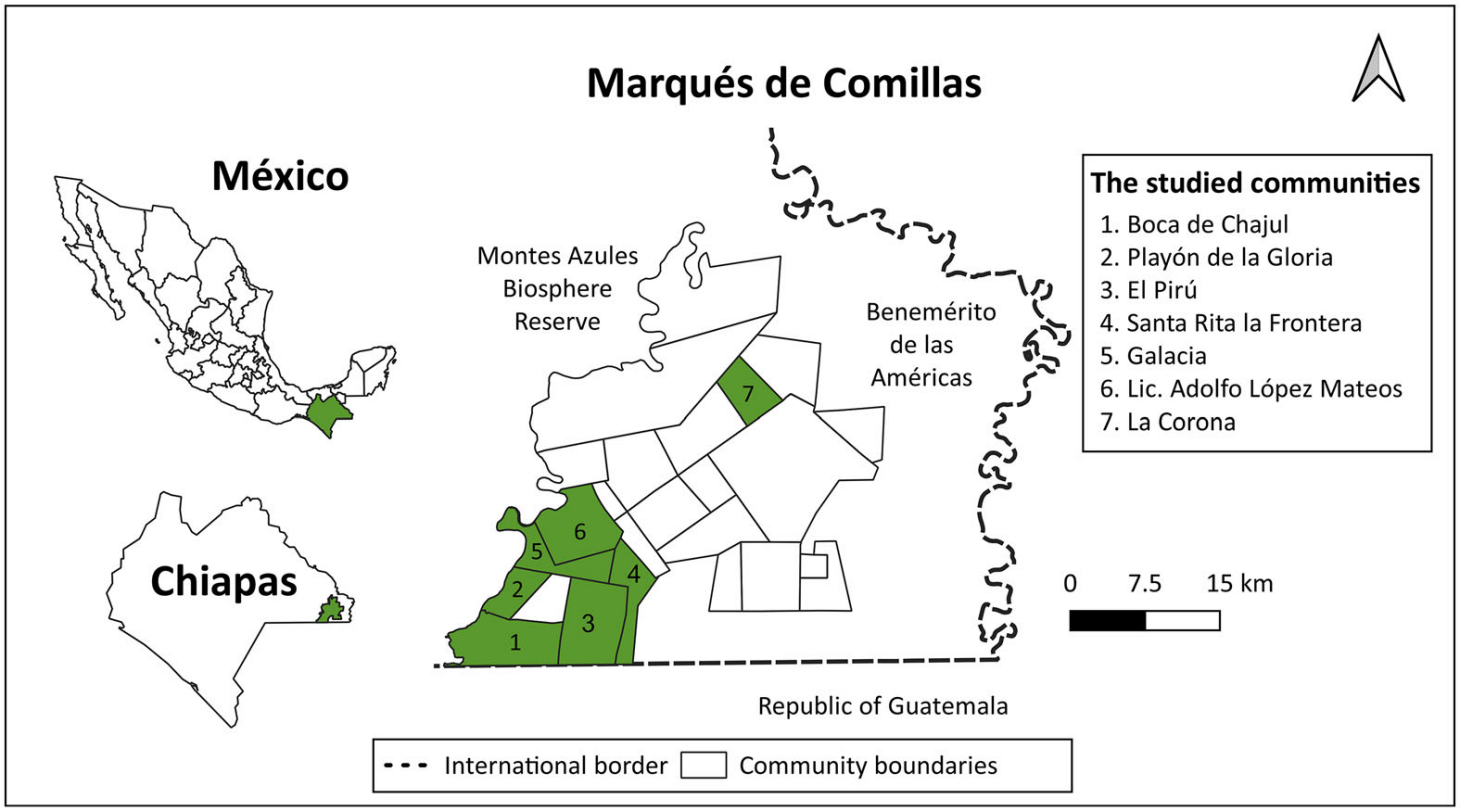
Study area in Selva Lacandona (Chiapas, Mexico), where participants in payments for ecosystem services (PES) programs were surveyed about perceptions and drivers of engagement related to biodiversity monitoring.

The 7 studied communities (ejidos) have participated in PES with advisory support from the NGO Natura y Ecosistemas Mexicanos since 2008 (Carabias et al., 2015) (Figure 1) (Table 2). Land enrollment has involved a combination of individual and communally held land plots. CONAFOR monitors compliance annually with remote sensing and field visits. Compliant participants receive an annual payment per hectare (currently set at MXN$1000 or ∼US$58). In the study area, sanctions for noncompliance include asking people to enroll additional lands to compensate for deforestation or removing deforested areas from the contracts (Izquierdo‐Tort et al., 2019). Previous research in MdC shows that PES programs have achieved high compliance levels (Izquierdo‐Tort et al., 2019) and avoided deforestation (Charoud et al., 2023; Costedoat et al., 2015).

**TABLE 2:** Key features of communities participating in payments for ecosystem services programs in Selva Lacandona (Chiapas, Mexico) surveyed about perceptions and drivers of engagement related to biodiversity monitoring.

Community name (year established)^a^	Total land area: number of hectares^a^	Population in 2020^b^	Main economic activities^a^	Number of PES contracts from 2008 to 2022^c^
Boca de Chajul (1976)	3810	385	Cattle ranching, agriculture	7
El Pirú (1980)	4984	207	Ecotourism, cattle ranching, agriculture	5
Galacia (1975)	2665	232	Ecotourism, cattle ranching, agriculture	6
La Corona (1985)	2251	330	Cattle ranching, agriculture	2
Licenciado Adolfo López Mateos (1979)	3012	281	Cattle ranching, agriculture	6
Playón de la Gloria (1974)	1739	210	Ecotourism, cattle ranching, agriculture	4
Santa Rita la Frontera (1985)	2402	220	Cattle ranching, agriculture	5

^a^ *Source*: Carabias et al. (2015).

^b^ *Source*: https://www.inegi.org.mx/programas/ccpv/2020/#Microdatos (accessed August 2023).

^c^ Data from Natura y Ecosistemas Mexicanos. The number of PES contracts in the period 2008–2022 considers only contracts with technical assistance provided by Natura y Ecosistemas Mexicanos.

We recruited 65 survey respondents from a population of 248 people in these communities who had enrolled individual land plots since 2008. We developed this list from PES enrollment data collected by NGO staff. We assessed survey respondents’ perceptions and interests related to biodiversity monitoring by conducting baseline and endline surveys during 1‐day training workshops in each community in May 2023. All eligible community members were invited to participate by their PES representative in an initial group meeting, where facilitators explained the terms of involvement. The 1‐day training workshop was held subsequently with those who volunteered. After administering a baseline survey, respondents received basic training in 4 monitoring tools: visual transects, camera traps, acoustic recorders, and satellite images of forest cover. We chose at least one tool from each category of observed‐based, earth‐based, and satellite‐based tools. The training consisted of 3 steps: overview of each tool; presentation of examples of data collected with each tool, namely, images showing forest cover change and data from camera traps and acoustic recorders previously collected by the NGO; and practice deploying and operating visual transects, camera traps, and acoustic recorders in a forest area in their community with guidance provided by workshop facilitators. An endline survey was administered after the training and the workshop concluded. In total, 65 people completed our baseline survey and participated in the workshops; 41 of those respondents also completed the endline survey. The surveys were completed individually in a digital tablet with support from workshop facilitators, taking care to ensure some level of physical separation between respondents.

The baseline survey contained demographic questions related to respondents’ age, sex, household size, total household income, main income sources, landholdings, and PES enrollment. The baseline and endline surveys contained the same set of questions around 3 themes that allowed us to compare responses before and after field engagement: monitoring interests and previous experience with monitoring tools; perceived advantages and disadvantages of monitoring tools; and expected outcomes and willingness to engage in biodiversity monitoring activities if introduced in PES. These themes were derived from previous scholarly literature (see “ADOPTING PARTICIPATORY BIODIVERSITY MONITORING APPROACHES IN PES”), which we adapted for PES particularities. Most survey questions were close‐ended and based on yes or no or Likert‐scale responses, which we analyzed quantitatively. We also included some open‐ended responses from which we drew verbatim quotes to illustrate our arguments through the respondents’ own words. Fieldwork activities were reviewed and approved by the ethics board at Posgrado en Ciencias de la Sostenibilidad at Universidad Nacional Autónoma de México. Survey questions are in Appendix S4.

## RESULTS

### Local willingness to engage and key respondent features

The 65 survey respondents represented 26% of the population who had enrolled in PES from 2008 to 2022 across the 7 studied communities and who lived in the region at the time of the survey (Table 3). Across communities, survey participation ranged from 11% to 56%. There was a positive association between participation in our study and previous PES engagement. Compared with the 183 PES participants who were not survey respondents, the 65 survey respondents had on average engaged in more PES contracts, enrolled more lands in each PES contract, and enrolled more land cumulatively in PES. These differences were statistically significant at a 5% significance level. Forty percent of survey respondents were women, which is a higher percentage than nonrespondents who participated in PES (36%), although this difference was not statistically significant.

**TABLE 3:** Key features of participants in payment for ecosystem services (PES) schemes in Selva Lacandona (Chiapas, Mexico) surveyed about perceptions and drivers of engagement related to biodiversity monitoring.

		Number of participants in PES scheme from 2008 to 2022^a^	Number of PES participants surveyed (% of PES participants)	Participants not surveyed (% of PES participants)	Test statistic (*p*), [confidence interval at 5% significance]
	Location				Not applicable
	Entire study region	248	65 (26)	183 (74)	
	Boca de Chajul	49	7 (14)	42 (86)	
	El Pirú	25	14 (56)	11 (44)	
	Galacia	36	8 (22)	28 (78)	
	La Corona	48	12 (25)	36 (75)	
	Licenciado Adolfo López Mateos	33	12 (36)	21 (64)	
	Playón de la Gloria	37	4 (11)	33 (89)	
	Santa Rita la Frontera	20	8 (40)	12 (60)	
	Sex				Chi‐square = 0.17 (0.6784), female [0.28–0.52], male [0.48–0.71]
	Female (%)	37	40	36	
	Male (%)	63	60	64	
Mean number of PES contracts from 2008 to 2022 (SD)		2.42 (1.33)	2.70 (1.23)	2.32 (1.36)	Welch *t* = 1.98 (0.0496^b^), [0.00067–0.76]
Mean ha of land enrolled in PES from 2008 to 2022 (SD)		53.07 (61.49)	81.87 (95.36)	43.19 (40.20)	Welch *t* = 2.99 (0.0039^c^), [12.91–64.45]
Average mean ha of land per PES contract from 2008 to 2022 (SD)		20.19 (19.36)	28.09 (29.98)	17.48 (13.05)	Welch *t* = 2.61 (0.0112^b^), [2.49–18.72]

^a^ Data retrieved from Natura y Ecosistemas Mexicanos A.C. The number of PES participants from 2008 to 2022 is only for contracts with technical assistance provided by the nongovernmental organization.

^b^ Significance at 5%.

^c^ Significance at 1%.

Respondents were mostly middle‐aged men (average age 52.9) (Appendix S1). The average household size was 3.8 and years of schooling was 6.5. On average, respondents reported owning 65.6 ha and an annual income in the previous year of MXN$204,000 (∼US$12,000 in 2023). Agriculture and cattle ranching were the main income sources, although ecotourism, wage labor, and public subsidies played a complementary role. There were large variations in contributions to PES schemes among households in terms of income and lands under contract (Appendix S1). Average reported PES annual payments were MXN$30,000 (∼US$1800 in 2023), which was <40% of total household income for almost 85% of respondents. Yet, >20% of respondents allocated >80% of their lands to PES.

### Previous monitoring experience and interests

One third of the respondents had previous experience with either camera traps (28%), visual transects (29%), or satellite images of forest cover (26%). Previous experience with specimen collection (15%), acoustic recorders (11%), lidar (5%), and drones (3%) was much lower. No respondent had previous e‐DNA experience.

Plants, birds, and mammals registered the highest interest among respondents, especially jaguar (*Panthera onca*), tapir (*Tapirella bairdii*), spider (*Atelles geofroyii*) and howler (*Alouatta pigra*) monkeys, white‐tailed deer (*Odocoileus virginianus*), collared peccary (*Dicotyles tajacu*), armadillo (*Dasypus novemcinctus*), spotted paca (*Cuniculus paca*), white‐nosed coati (*Nasua narica*), agouti (*Dasyprocta punctata*), bats (Chiroptera), scarlet macaw (*Ara macao cyanoptera*), toucans (*Ramphastos sulfuratus*), trogon (*Trogon* sp.), great curassow (*Crax rubra*), orchids (Orchidaceae), mahogany (*Swietenia macrophylla*), cedar (*Cedrela odorata*), medicinal plants, heliconia (*Heliconia* sp.), pitaya (Cactaceae), kapok tree (ceiba) (*Ceiba pentandra*), and ironwood (guapaque) (*Dialium*) (Table 4). Amphibians, reptiles, and insects were the least popular groups, although some respondents mentioned butterflies and crocodiles. In terms of monitoring tools, visual transects, camera traps, and drones registered the highest levels of interest, followed by forest cover satellite images and acoustic recorders. Specimen collection, lidar, and e‐DNA were the least popular.

**TABLE 4:** Interests in monitoring indicators and methods among participants in payments for ecosystem services programs in Selva Lacandona (Chiapas, Mexico) surveyed about perceptions and drivers of engagement related to biodiversity monitoring.

Monitoring indicator and tool		Total sample (*n* = 65)	Responses depending on previous monitoring experience with key tools for all respondents (*n* = 65)																				Responses for cohort that completed baseline and endline surveys (*n* = 41)				
			Visual transects					Remote sensing (forest cover)					Camera traps				Acoustic recorders										
			Yes (*n* = 19)	No (*n* = 46)	Comparison (Mann–Whitney)			Yes (*n* = 17)	No (*n* = 48)	Comparison (Mann–Whitney)			Yes (*n* = 18)	No (*n* = 47)	Comparison (Mann–Whitney)		Yes (*n* = 7)		No (*n* = 58)	Comparison (Mann–Whitney)^a^			Baseline (*n* = 41)	Endline (*n* = 41)	Comparison (Wilcoxon signed rank)		
		Mean (SD)	Mean (SD)	Mean (SD)	*U*	*p*	CI	Mean (SD)	Mean (SD)	*U*	*p*	CI	Mean (SD)	Mean (SD)	*U* stat	*p*	CI	Mean (SD)	Mean (SD)	*U*	*p*	CI	Mean (SD)	Mean (SD)	*W*	*p*	CI
Indicator	Deforestation	3.1 (1)	3.1 (1)	3.1 (0.9)	421.5	0.839	−1, 1	3.4 (0.9)	3 (1)	448.5	0.148	0, 1	3.3 (1)	3 (1)	461	0.286	0, 1	3.3 (1)	3.1 (1)	216.5	0.577	−1, 1	3.1 (0.9)	3.3 (0.9)	94	0.173	−0.2, 0.6
	Mammals	3.5 (0.8)	3.8 (0.5)	3.4 (0.9)	518	0.045^*^	0, 1	3.8 (0.4)	3.3 (0.9)	488.5	0.033^*^	0, 1	3.9 (0.3)	3.3 (0.9)	548	0.006^**^	0, 1	4.0 (0)	3.4 (0.9)	NA	NA	NA	3.5 (0.8)	3.6 (0.8)	45	0.388	−2, 0.4
	Birds	3.4 (0.9)	3.6 (1)	3.3 (0.8)	534	0.042^*^	0, 1	3.8 (0.4)	3.2 (1)	522.5	0.013^*^	0, 1	3.8 (0.7)	3.2 (0.9)	569	0.005^**^	0, 1	4.0 (0)	3.3 (0.9)	NA	NA	NA	3.6 (0.7)	3.7 (0.7)	25	0.486	−0.2, 0.3
	Reptiles	2.2 (1.3)	2.4 (1.3)	2.2 (1.2)	475.5	0.462	−1, 2	2.2 (1.3)	2.3 (1.2)	374	0.895	−2, 1	3 (1.2)	1.9 (1.1)	607.5	0.001^**^	0.5, 3	2.7 (1.4)	2.1 (1.2)	245.5	0.259	−1, 3	2.1 (1.2)	2.6 (1.2)	28.5	0.003^**^	0.3, 0.9
	Amphibians	2.2 (1.1)	2.3 (1.2)	2.1 (1.1)	468.5	0.531	−1, 2	2.4 (1.2)	2.1 (1.1)	440	0.345	−1, 2	2.5 (1.2)	2 (1.1)	503.5	0.116	−1, 2	2.6 (1.4)	2.1 (1.1)	239	0.327	−1, 3	2 (1.1)	2.7 (1.2)	51	0.002^**^	0.3, 1
	Fish	3.1 (1)	3.1 (1.2)	3.2 (1)	423.5	0.956	−1, 1	3.3 (1)	3 (1)	441.5	0.321	−1, 1	3.2 (1.1)	3.1 (1)	433	0.652	−1, 1	3.6 (0.8)	3.1 (1)	252	0.191	0, 1	3.2 (1)	3.2 (1.1)	136	0.962	−0.4, 0.3
	Insects	2.5 (1.2)	2.6 (1.1)	2.4 (1.2)	462	0.499	−1, 1	2.5 (1.1)	2.5 (1.2)	370.5	0.960	−1, 1	2.9 (1.1)	2.3 (1.1)	516	0.054	0.5, 2	2.7 (1.3)	2.4 (1.1)	220	0.533	−2, 2	2.4 (1.2)	2.8 (1.3)	54	0.049^*^	0, 0.7
	Plants	3.6 (0.8)	3.6 (1)	3.5 (0.8)	478.5	0.362	0, 0	3.9 (0.3)	3.4 (0.9)	495.5	0.031^*^	0, 1	3.9 (0.3)	3.4 (0.9)	527	0.024^*^	0, 1	4.0 (0)	3.5 (0.9)	NA	NA	NA	3.6 (0.7)	3.8 (0.5)	19.5	0.049^*^	0, 0.5
	Fungi	3 (1.1)	2.9 (1.2)	3 (1.2)	409	1	−1, 1	3.1 (1.2)	2.9 (1.2)	403.5	0.513	−1, 1	3.2 (1.2)	2.8 (1.2)	447.5	0.312	−1, 1	2.9 (1.2)	2.9 (1.2)	181	0.857	−2, 1	3.1 (1.2)	3.2 (1)	60.5	0.272	−0.2, 0.6
Tool	Specimen collection	2.5 (1.2)	2.4 (1.3)	2.5 (1.2)	360	0.770	−1.5, 1.5	2.8 (1.3)	2.3 (1.2)	419.5	0.211	−1, 2	2.8 (1.3)	2.3 (1.2)	432.5	0.258	−1, 2	2.6 (1.4)	2.5 (1.2)	196.5	0.802	−2, 2	2.5 (1.3)	2.7 (1.3)	16.5	0.020^*^	0.1, 0.7
	Visual transects	3.5 (0.8)	3.9 (0.3)	3.4 (0.9)	577.5	0.014^*^	0, 1	3.8 (0.4)	3.4 (0.9)	455.5	0.233	0, 0.5	3.9 (0.2)	3.3 (0.9)	574	0.004^**^	0, 1	3.9 (0.4)	3.5 (0.9)	244	0.252	0, 0	3.6 (0.8)	3.6 (0.7)	42.5	0.533	−0.2, 0.4
	Remote sensing (forest cover)	3.0 (1.1)	3.3 (1)	2.9 (1.1)	402	0.184	−0.5, 1	3.5 (0.6)	2.8 (1.2)	429	0.058	−0.5, 1.5	3.5 (0.9)	2.8 (1.1)	497	0.025^*^	0, 1	3.1 (1.2)	3.0 (1.1)	198.5	0.689	−2, 1	2.9 (1.1)	3.5 (0.7)	12	0.006^**^	0.4, 1.1
	Lidar	2.4 (1.2)	2 (1.2)	2.6 (1.2)	78.5	0.219	−2, 1	2.7 (1.3)	2.4 (1.2)	117	0.569	−2, 2	2.4 (1.4)	2.5 (1.2)	116.5	0.873	−2, 2	1.8 (1.5)	2.5 (1.2)	39	0.285	−2, 1	2.3 (1.3)	2.3 (1.2)	7	1.0	−0.7, 0.8
	Camera traps	3.2 (1.2)	3.6 (1)	3 (1.2)	554	0.056	0, 1	3.9 (0.3)	2.9 (1.2)	565	0.002^**^	0, 1.5	3.9 (0.2)	3 (1.2)	614.5	0.001^**^	0, 1	4.0 (0)	3.1 (1.1)	NA	NA	NA	3.3 (1.1)	3.7 (0.7)	33	0.038^*^	0, 0.8
	Drones	3.2 (1.2)	3.6 (0.9)	3 (1.2)	523.5	0.048^*^	0, 1	3.4 (1.1)	3.1 (1.2)	422.5	0.293	−1, 1	3.8 (0.7)	2.9 (1.2)	559.5	0.004^**^	0, 1.5	4.0 (0)	3.1 (1.2)	NA	NA	NA	3.1 (1.2)	3.6 (0.9)	24.5	0.013^*^	0.2, 1
	Acoustic recorders	3 (1.2)	2.9 (1.2)	3 (1.1)	424	0.848	−1.5, 1	3.5 (0.6)	2.8 (1.2)	527.5	0.026^*^	0, 1.5	3.3 (1)	2.9 (1.2)	484.5	0.265	−1, 1	3.7 (0.8)	2.9 (1.1)	286.5	0.047^*^	0, 1	3 (1.1)	3.5 (0.8)	88	0.021^*^	0.1, 0.8
	e‐DNA	2.3 (1.3)	2.2 (1.3)	2.3 (1.3)	114	0.819	−2, 2	2.7 (1.3)	2 (1.3)	141.5	0.177	−1, 3	2.4 (1.4)	2.2 (1.2)	125.5	0.686	−2, 3	2.0 (1.4)	2.3 (1.3)	51	0.784	−2, 2	1.9 (1.2)	2.1 (1.2)	10.5	0.605	−0.6, 1.3

*Note*: Responses in Likert scale to question “How interested are you in monitoring the following?” are as follows: 1 = *strong no*; 2 = *slight no*; 3 = *slight yes*; and 4 = *strong yes*. * and ** represent significance at 5% and 1%, respectively.

^a^ Result not available (NA).

On average, respondents with previous monitoring experience registered higher interest in using various tools than respondents without experience (Table 4). The same applied for specific indicators. Many of these differences were statistically significant (Table 4).

### Willingness to engage in monitoring and key respondent characteristics

**FIGURE 2:**
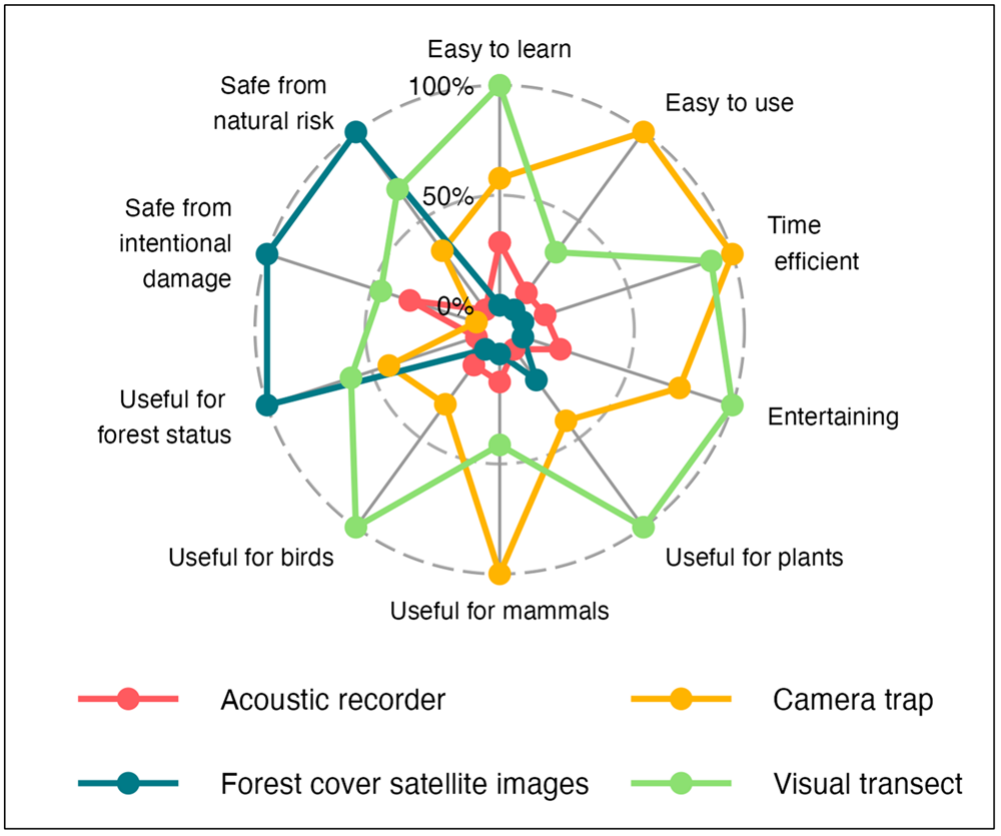
Ranking of preference for visual transects, camera traps, acoustic recorders, and forest cover satellite images relative to key criteria according to baseline survey respondents (*n* = 65) in Selva Lacandona (Chiapas, Mexico) participating in payments for ecosystem services (PES) programs and surveyed about perceptions and drivers of engagement related to biodiversity monitoring. Respondents were asked to rank the 4 tools from 1 to 4 for each criterion (4, highest; 1, lowest). The value on each axis is normalized to the mean of the best ranked tool in each criterion.

For the 65 respondents to the baseline survey, visual transects and camera traps placed first or second across 7 of 10 categories, including ease of learning, ease of use, time efficiency, entertainment, and usefulness for monitoring plants, mammals, and birds (Figure 2). Although forest cover satellite images fared poorly across these categories, it ranked first in the 3 remaining categories: usefulness for monitoring forest status, safety from natural damage, and safety from intentional damage. Camera traps ranked last in terms of safety from intentional damage; respondents stated the presence of illegal activities related to poaching as the major risk of theft of or tampering with equipment. Acoustic recorders ranked poorly across all categories.

Twenty‐four respondents completed only the baseline survey and participated in the workshops but did not wish to complete the endline survey. This group expressed fatigue due to previous participation in research projects conducted in the study area. Their demographic characteristics did not differ statistically from the group that completed both baseline and endline surveys, except for household size (Appendix S2).

For the 41 respondents who completed both surveys, interest in all monitoring indicators was higher at the endline survey than the baseline survey (Table 4). There were statistically significant differences for reptiles, amphibians, insects, and plants. Reptiles, amphibians, and plants had the lowest interest registered at baseline. Interest was higher at the endline survey than the baseline survey for all monitoring tools. There were significant differences for camera traps, acoustic recorders, forest cover satellite images, drones, and specimen collection (Appendix S3). There were few differences between baseline and endline surveys in ranking of monitoring tools.

Seventeen respondents to the endline survey were positively surprised that camera trap records revealed a higher‐than‐expected presence and diversity of animal species. Respondents were excited to see certain endangered animals that were unknown or thought to no longer be present on the community lands. A respondent from Playón de la Gloria said, “To see the animals up close like the tapir or jaguar. These are animals that are hard to see up close and with this [the camera] you can see what they do, when they walk, and what they do at night.” In turn, 16 respondents were negatively surprised that satellite images showed a higher‐than‐expected level of deforestation in their community. A respondent from Santa Rita la Frontera said, “There was a lot of forest before. I did not imagine that so much was cut down.”

### Perceived benefits and willingness to engage

**FIGURE 3:**
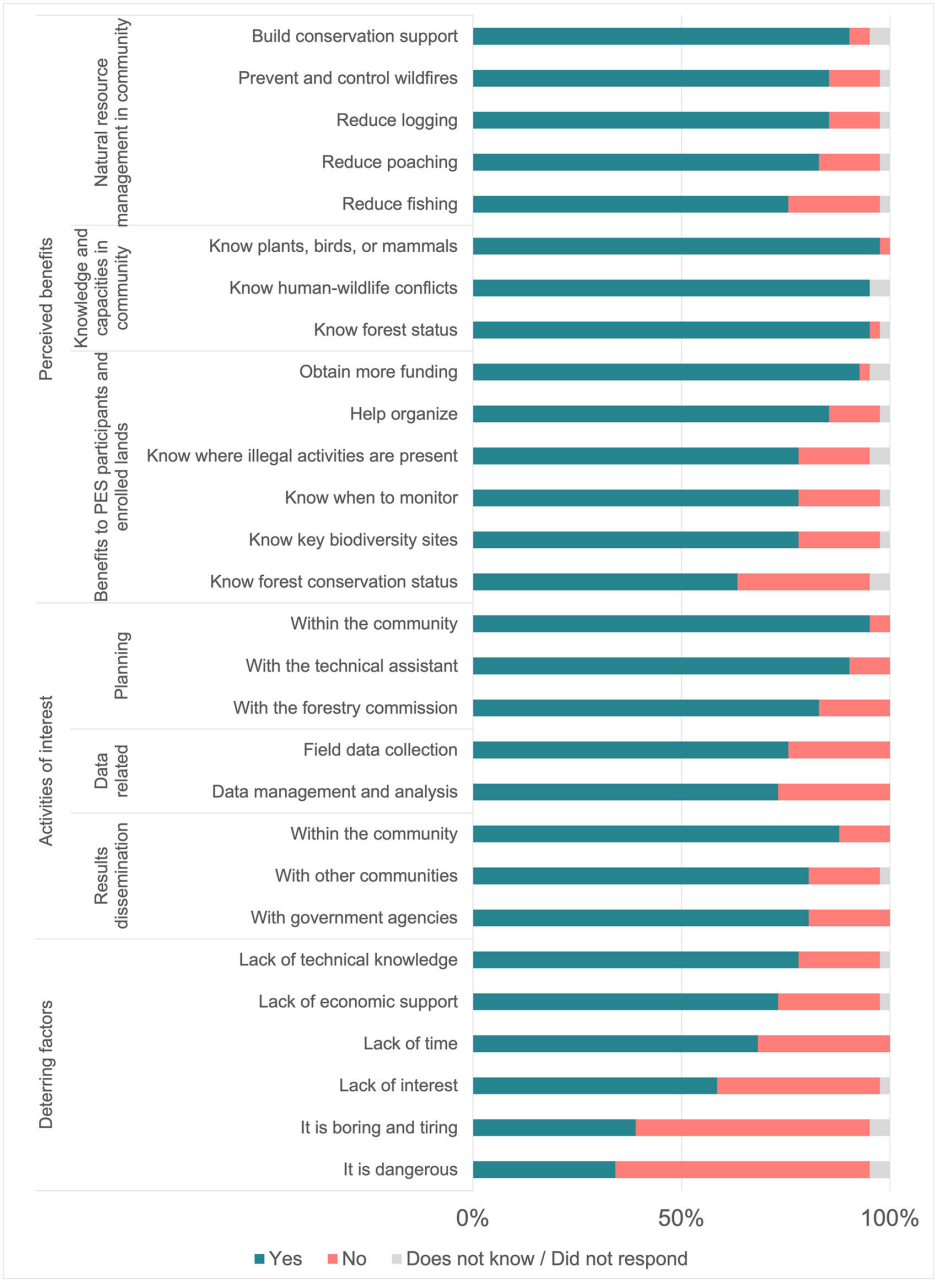
Perceived benefits of and deterrents to monitoring of biodiversity in payments for ecosystem services (PES) programs and activities of interest according to endline respondents (*n* = 41) to a survey on perceptions and drivers of engagement related to biodiversity monitoring among PES participants in Selva Lacandona (Chiapas, Mexico).

The majority of respondents who completed the endline survey perceived that introducing biodiversity monitoring in a PES context could yield multiple benefits, including contributions to knowledge related to biodiversity and forest conservation status in‐ and outside PES lands, human–wildlife conflicts (e.g., livestock predation by jaguars), and the best places and times to conduct activities; contributions to natural resource management related to the reduction of illegal activities (i.e., logging, poaching, and fishing), wildfire prevention and control, and collective planning and organization; and building conservation support within the community and the possibility of increasing PES funding (Figure 3).

There was a high level of interest in participating in monitoring activities if introduced into PES in the future (Figure 3). The highest rate of positive responses (above 80%) was for participating in planning of monitoring activities (i.e., meetings to organize the execution of biodiversity monitoring activities) and dissemination of results, particularly within respondents’ own communities. Field data collection activities and managing and analyzing the data collected—including tasks such as data storage, processing, examination, interpretation, and visualization—had fewer positive responses, but these were still above 70%.

When asked about potential factors that could deter future engagement in biodiversity monitoring (Figure 3), lack of time, interest, and technical and economic support (which could include lack of payments or not enough economic income in the household) received positive responses from ∼60–80% of respondents. Boredom, fatigue (i.e., getting bored or tired from continued engagement), and risk to personal security received positive responses from ∼35–40% of respondents.

## DISCUSSION

To our knowledge, we are the first to explore how participatory biodiversity monitoring can be adopted in a PES context, which adds to the limited body of research directly addressing PES’ biodiversity outcomes (Bremer et al., 2019; Chen et al., 2020). We considered our findings in relation to the literature on local drivers of engagement and perceptions in environmental monitoring and PES enrollment.

### Willingness to engage

Overall, ∼25% of current PES participants across the 7 studied communities participated in our surveys and workshops, which is more than in previous studies assessing local willingness to engage in monitoring activities (e.g., 2–14% in Brites & Morsello [2018]). Yet, we are cautious in equating this rate of engagement with the level of interest in monitoring because many people mentioned previous participation in recent research projects involving local data collection as the main reason for opting out. Additionally, engagement in our study may signal some interest in monitoring but not necessarily commitment to engage in monitoring activities.

Economic reliance on PES schemes (i.e., PES contributions to total income and land amounts enrolled to PES) at the household level was positively associated with volunteering to participate in our study, and lack of economic support was as a key deterrent for future engagement. Both findings are consistent with previous studies that show those who benefit more from natural resources contribute the most to their conservation (Brites & Morsello, 2018; Dalton et al., 2012). Lack of technical knowledge, time, and personal interest in monitoring were other deterrents for most respondents, which is consistent with the literature on the monetary and nonmonetary nature of drivers of monitoring interest (Brites & Morsello, 2018).

Boredom and fatigue and personal security risks were also determents to participation in biodiversity monitoring activities (listed by ∼35–40% of respondents), as noted previously in other non‐PES contexts (Gabillet et al., 2020; Soller et al., 2020; Tomaszewski & Kołakowski, 2023). Our tool selection may have influenced results because deployment of visual transects, camera traps, and acoustic recorders is time consuming and repetitive. We found that the mention by respondents of potential personal security risks associated with monitoring was mostly related to the presence of poachers and illegal wildlife traffickers in the study area, which PES participants avoid encountering in their lands and are reluctant to denounce due to fear of reprisal. In such contexts, local engagement in monitoring is crucial to secure participants’ safety and identify and minimize risks throughout the monitoring process (Sandbrook et al., 2021).

### Local monitoring interests and perceptions

Our study respondents demonstrated a particular affinity for monitoring plants, birds, and mammals and little interest in monitoring amphibians, reptiles, and insects. The species named by respondents, such the jaguar and the scarlet macaw, are consistent with the literature showing a taxonomic bias toward species that are more charismatic, locally abundant, or easily identified (Monsarrat & Kerley, 2018). Although monitoring capacities and priorities among local populations may not always match global biodiversity conservation needs (Collen et al., 2013; Wyborn & Evans, 2021), involving local people in monitoring activities and decision‐making processes (Becker et al., 2005; Danielsen et al., 2021) and gathering evidence on local people's perceptions (Bennett, 2016) can increase local participation and provide critical insights for improving biodiversity monitoring approaches.

Visual transects and camera traps, which ranked first according to respondents for monitoring mammals, birds, and plants, were the 2 most preferred tools. Yet, drones were also intriguing to respondents, despite low levels of previous experience, which can be attributed to their ability to provide precise and up‐to‐date spatial information on territorial impacts to address land conflicts or to prepare for natural disasters (Vargas‐Ramírez & Paneque‐Gálvez, 2019). Acoustic recorders ranked poorly in all categories despite increased uptake and interest as a monitoring tool among researchers, decision makers, and managers (Eldridge et al., 2018).

Visual transects and camera traps scored the highest in 7 out of 10 ranking categories (i.e., ease of learning, ease of using, time efficiency, entertainment, and usefulness for monitoring plants, mammals, and birds), whereas forest cover satellite images ranked first in usefulness for monitoring forest status and safety from both natural or intentional damage. This confirms that productive monitoring synergies should be built by deploying multiple tools simultaneously because they produce complementary insights and information (Mulatu et al., 2017; Nuñez et al., 2019).

### Exposure effects in biodiversity monitoring

Almost 30% of study respondents had previous experience with either visual transects, camera traps, or forest cover satellite images through engagement in monitoring activities developed by a local NGO that focus on monitoring large mammals and scarlet macaws (Carabias et al., 2015). Although this situation may not occur in other contexts, our results shed some light on how previous exposure can influence subsequent interest in monitoring.

The higher levels of interest in monitoring we observed at baseline among study respondents with previous experience can be attributed to self‐selection (i.e., those with higher predisposition for monitoring were more likely to have done so before our study) or an exposure effect (i.e., exposure to a stimulus, in this case biodiversity monitoring activities, increases subsequent interest and attitudes) (Zajonc, 1968). Yet, higher reported interest in monitoring at endline than baseline for the same respondents is consistent with a positive exposure effect. Exposure effects have been studied and documented in several fields of study (Montoya et al., 2017) but have received scant attention in conservation science. These positive effects may have been associated with respondents’ reactions to receiving new insights from their communities, such as satellite images showing higher‐than‐expected deforestation levels. Future research could explore drivers of exposure effects because they can provide clues for sustaining local monitoring interests over time.

### Policy implications

We believe our findings support incorporating participatory biodiversity monitoring into PES, with some caveats. The rate of willingness to engage in our study's activities among current PES participants was ∼25%, but research shows that a few motivated volunteers in a community are sufficient for achieving monitoring tasks (Norris et al., 2018). Further, our respondents demonstrated high interest in applying various tools to monitor different indicators and stated their willingness to participate in diverse monitoring activities in the future. They also identified several benefits of introducing biodiversity monitoring into PES, such as enhancing knowledge and skills, natural resource management, and conservation support within the community (Becker et al., 2005; Fernandez‐Gimenez et al., 2008; Krause & Zambonino, 2013; Newman et al., 2003; Rakotomahazo et al., 2019; Schröter et al., 2018; Sterling et al., 2017; Upton, 2020).

Our finding that the willingness to engage in monitoring depends on economic (i.e., PES’ contributions to total income and land amounts enrolled to PES) and noneconomic factors (i.e., boredom and fatigue) implies the need to identify suitable incentives for each local context to achieve and sustain involvement over time. Our respondents voluntarily participated without monetary compensation, but it is unclear if such involvement could be maintained throughout a PES contract or if new volunteers could be recruited when others opt out (Brites & Morsello, 2018). Although providing monetary compensation for monitoring could help increase individual participation levels, reliance on payments could crowd out prosocial or environmental motivations (Ezzine‐de‐Blas et al., 2019; Lliso et al., 2022; Rode et al., 2015), lead to capture of benefits (Izquierdo‐Tort et al., 2022; Milne & Adams, 2012), and threaten long‐term sustainability if funding is scarce (Brites & Morsello, 2018). The literature suggests that providing nonmonetary benefits (e.g., in‐kind payments, skills development) or promoting intrinsic environmental values could also help increase engagement (Norris et al., 2018). Future research could explore whether cost‐sharing arrangements with parties interested in biodiversity monitoring, such as research centers, NGOs, or international organizations, could help PES implementers cover monitoring costs and ensure the transfer of technical knowledge and other requirements without imposing large burdens on PES participants or implementers (Stephenson et al., 2017).

Our results indicating uneven levels of interest in monitoring among local PES participants and preferences for specific tools and indicators have implications for data reliability when monitoring biodiversity in a PES context. First, spatial bias in data collection could result if landholders monitor their own land and not all people participate (Dickinson et al., 2010). Self‐monitoring could also produce conflict of interest if payments are conditional on reported biodiversity levels (Sommervill et al., 2010). Both issues could be mitigated by external third‐party monitoring, but such benefits should be weighed against the loss of contextual knowledge, limited learning and motivational aspects, and potential ethical and logistical issues related to data access and ownership (Sandbrook et al., 2021). Second, the strong preference and affinity for certain species of taxonomic groups, such as large mammals, could produce bias if certain indicators are omitted or overrepresented (Monsarrat & Kerley, 2018). This fact, together with previous findings that participatory monitoring and enforcement approaches in PES are not always effective (Eisenbarth et al., 2021; Naime et al., 2022), suggests that attention should be paid to local preferences, norms, and institutions when devising monitoring rules. This matters because Mexico's PES experience shows that standardizing required activities across contexts with limited local input imposes significant costs for participants in terms of time, labor, and money (Alix‐Garcia et al., 2018; Avila‐Foucat et al., 2021; Izquierdo‐Tort et al., 2021; Jones et al., 2018).

We identified 3 limitations of our study. First, our small size was sample and extracted from a contextualized intervention that relied mostly on survey data collection, which limits the consequentiality of some responses, such as those related to willingness to engage, and the generalizability of findings to other settings. However, our survey results are consistent with a wider pattern of male land ownership, low educational attainment, large land endowments, and agricultural‐based livelihoods coexisting with high PES enrollment rates in the municipality of Marqués de Comillas (Izquierdo‐Tort, 2020; Izquierdo‐Tort et al., 2021, 2019, 2022). Also, the theoretical and methodological approaches and findings can be applied to other situations where PES is expected to contribute to biodiversity conservation. Further research could explore future willingness to engage in monitoring in a PES context through discrete choice experiments (Canessa et al., 2023; Vorlaufer et al., 2023). Second, we tested local engagement and perceptions regarding monitoring over short periods in a field research context; thus, it is unclear whether our results would hold over longer periods or in an actual PES program setting. Finally, we field tested a few monitoring tools that are accessible and relevant for our study area; thus, our results do not cover the full spectrum of tools.

PES programs have been criticized for not monitoring impacts on ecosystem services, particularly biodiversity. Our results showed potential for deploying and adapting participatory biodiversity monitoring approaches in PES schemes, with some caveats. As the global biodiversity crisis continues (Giam, 2017; Gibson et al., 2011) and public support and funding for some PES schemes is eroding (Etchart et al., 2020; Hayes et al., 2022; Izquierdo‐Tort et al., 2021; Wunder et al., 2018), we hope our study can provide relevant insights for researchers and practitioners interested in understanding and improving PES’ potential for biodiversity conservation.

## Supporting information

Supplementary information
