# Supplementary material for: Exploring local perceptions and drivers of engagement in biodiversity monitoring among participants in payments for ecosystem services schemes in southeastern Mexico
Source: Conserv Biol. 2024 Apr 25;38(6):e14282. doi: 10.1111/cobi.14282 (PMC11588995; doi:10.1111/cobi.14282)
Supplement: Supplementary file 1 — Supplementary information [file COBI-38-e14282-s001.docx]

**Supplementary information:** Exploring local perceptions and drivers of engagement in biodiversity monitoring among participants in payments for ecosystem services schemes in southeastern Mexico

**Appendix S1.**

| Table S1. Key characteristics of survey respondents participating in payments for ecosystem services programs in  Selva Lacandona (Chiapas, Mexico) surveyed about perceptions and drivers of engagement related to biodiversity  monitoring. | | | | | | | | | | |
| --- | --- | --- | --- | --- | --- | --- | --- | --- | --- | --- |
| Variable | | Unit | Total study sample (*n*=65) | Community | | | | | | |
|  |  |  |  | Lic. Adolfo Lopez Mateos  (*n*=12) | Boca de Chajul  (*n*=7) | El Piru  (*n*=14) | Galacia  (*n*=8) | La Corona  (*n*=12) | Playon de la Gloria  (*n*=4) | Santa Rita la Frontera  (*n*=8) |
| Age | | Mean | 52.9 | 56.0 | 52.0 | 49.1 | 55.9 | 55.7 | 63.0 | 43.7 |
|  |  | SD | 12.9 | 12.6 | 13.1 | 11.8 | 12.3 | 13.9 | 16.8 | 8.6 |
| Sex | Female | % | 40.00% | 33.33% | 71.43% | 35.71% | 50.00% | 33.33% | 0.00% | 50.00% |
|  | Male | % | 60.00% | 66.67% | 28.57% | 64.29% | 50.00% | 66.67% | 100.00% | 50.00% |
| School years | | Mean | 6.5 | 6.8 | 8.4 | 6.5 | 7.6 | 5.2 | 3.2 | 7.2 |
|  |  | SD | 4.0 | 4.8 | 2.5 | 3.6 | 3.4 | 4.1 | 4.3 | 4.7 |
| Household size | | Mean | 3.8 | 3.8 | 5.0 | 2.9 | 4.7 | 3.9 | 2.2 | 3.9 |
|  |  | SD | 1.9 | 1.8 | 2.8 | 1.6 | 2.5 | 1.6 | 0.5 | 1.5 |
| Total lands in hectares | | Mean | 65.6 | 87.8 | 21.2 | 79.2 | 73.4 | 53.6 | 29.2 | 71.4 |
|  |  | SD | 52.4 | 30.1 | 15.4 | 74.5 | 20.2 | 51.6 | 19.8 | 62.2 |
| Monthly household income | | Mean | 14,083.4 | 19,022.2 | 9,515.0 | 20,035.7 | 12,614.3 | 5,000.0 | 5,300.0 | 7,250.0 |
|  |  | SD | 22,747.8 | 24,004.9 | 6,340.1 | 35,046.9 | 17,253.3 | 4,242.6 | 1,135.8 | 5,326.8 |
| Yearly household income in previous year | | Mean | 204,813.5 | 245,000.0 | 146,694.3 | 325,028.6 | 175,357.1 | 93,000.0 | 66,200.0 | 95,883.3 |
|  |  | SD | 359,799.6 | 204,605.7 | 61,896.8 | 632,729.5 | 187,575.5 | 4,242.6 | 17,998.5 | 62,844.7 |
| Yearly income from PES | | Mean | 29,896.9 | 23,166.7 | 10,285.7 | 56,885.7 | 35,312.5 | 24,181.8 | 6,875.0 | 23,875.0 |
|  |  | SD | 30,566.1 | 21,787.6 | 7,674.9 | 43,817.2 | 14,295.3 | 28,205.7 | 3,705.3 | 18,749.8 |
| PES yearly income as % of total household income | 0% | % | 12.50% | 25.00% | 28.57% | 0.00% | 0.00% | 27.27% | 0.00% | 0.00% |
|  | 1-20% |  | 46.87% | 41.66% | 57.14% | 35.71% | 37.50% | 36.36% | 100.00% | 62.50% |
|  | 21-40% |  | 25.00% | 25.00% | 14.28% | 42.85% | 25.00% | 18.18% | 0.00% | 25.00% |
|  | 41-60% |  | 9.37% | 0.00% | 0.00% | 7.14% | 37.50% | 18.18% | 0.00% | 0.00% |
|  | 61-80% |  | 3.12% | 8.33% | 0.00% | 7.14% | 0.00% | 0.00% | 0.00% | 0.00% |
|  | 81-100% |  | 3.12% | 0.00% | 0.00% | 7.14% | 0.00% | 0.00% | 0.00% | 12.50% |
| PES enrolled lands as as % of total household lands | 0% | % | 9.37% | 16.66% | 28.57% | 0.00% | 0.00% | 0.00% | 0.00% | 25.00% |
|  | 1-20% |  | 14.06% | 33.33% | 0.00% | 7.14% | 12.50% | 9.09% | 25.00% | 12.50% |
|  | 21-40% |  | 23.43% | 16.66% | 0.00% | 14.28% | 25.00% | 45.45% | 50.00% | 25.00% |
|  | 41-60% |  | 17.18% | 25.00% | 28.57% | 7.14% | 37.50% | 9.09% | 0.00% | 12.50% |
|  | 61-80% |  | 14.06% | 8.33% | 14.28% | 7.14% | 25.00% | 27.27% | 0.00% | 12.50% |
|  | 81-100% |  | 21.87% | 0.00% | 28.57% | 64.28% | 0.00% | 9.09% | 25.00% | 12.50% |

**Appendix S2.**

| Table S2. Comparison of responses between survey respondents who completed the baseline survey exclusively  (*n*=24) and those who completed both the baseline and endline survey (*n*=41). Surveys focused on perceptions and  drivers of engagement related to biodiversity monitoring conducted among participants in payments for ecosystem  services programs in Selva Lacandona (Chiapas, Mexico). | | | | | | | | | |
| --- | --- | --- | --- | --- | --- | --- | --- | --- | --- |
| Variable | Total sample (*n*=65) | | Both endline and baseline respondents (*n*=41) | | Only baseline respondents (*n*=24) | | Comparison between both survey respondents and only endline respondents | | |
|  | Mean | SD | Mean | SD | Mean | SD | Test Statistic^a^ | *p* | CI |
| Age | 52.95 | 12.94 | 52.19 | 11.83 | 54.25 | 14.82 | 513.5 | 0.775 | [-6.5,  10] |
| Sex | Male = 60% Male = 40% | | Male = 56%  Male = 44% | | Male = 67% Female = 33% | | 0.33 | 0.563 | n.a. |
| School years | 6.51 | 4.01 | 6.27 | 3.81 | 6.9167 | 4.38 | 550.5 | 0.416 | [0, 6] |
| Household size | 3.78 | 1.93 | 3.39 | 1.74 | 4.46 | 2.08 | 642 | 0.037^b^ | [0, 2] |
| Total lands | 65.58 | 52.44 | 63.74 | 55.90 | 68.69 | 47.02 | 499 | 0.466 | [-3,  53.02] |
| Monthly income | 14083.44 | 22747.78 | 14,841.13 | 25,803.93 | 12,701.76 | 16,384.35 | 278 | 0.762 | [-2,500,  5,500] |
| Yearly household income in previous year | 204,813.47 | 359,799.6 | 222,040.62 | 442,254.1 | 172,385.88 | 87,768.93 | 359.5 | 0.067 | [6,100, 162,700] |
| Yearly income from PES | 29,896.87 | 30,566.08 | 31,002.5 | 33,102.85 | 28,054.17 | 26,369.79 | 458.5 | 0.770 | [-16,000, 15,512.5] |
| ^a^Test of comparison based on Mann-Whitney *U* test for all variables except for Sex where *Chi-squared* test was  applied.  ^b^Significance at 5%.  ^c^Significance at 1%. | | | | | | | | | |

**Appendix S3.**

| Comparison of ranking responses for respondents who completed both baseline and endline surveys  (n=41). Surveys focused on perceptions and drivers of engagement related to biodiversity  monitoring conducted among participants in payments for ecosystem services programs in Selva  Lacandona (Chiapas, Mexico). | | | | | | | | |
| --- | --- | --- | --- | --- | --- | --- | --- | --- |
| Criteria | Tool | Baseline responses^a^ | | Endline responses^a^ | | Comparison (paired T test excluding missing paired data) | | |
|  |  | Mean | SD | Mean | SD | Test stat | *p* | CI |
| Easy to learn | Camera trap | 1.97 | 0.98 | 1.97 | 0.72 | -0.15 | 0.878 | [1.65 ,2.29] |
|  | Acoustic recorder | 2.69 | 0.79 | 2.58 | 0.80 | 0.47 | 0.637 | [2.43 ,2.95] |
|  | Visual transect | 1.74 | 0.90 | 1.53 | 0.77 | 1.81 | 0.076 | [1.44 ,2.03] |
|  | Forest cover satellite image | 3.58 | 0.75 | 3.90 | 0.30 | -2.76 | 0.008^c^ | [3.34 ,3.83] |
| Easy to use | Camera trap | 1.51 | 0.75 | 1.58 | 0.77 | -0.58 | 0.562 | [1.26 ,1.75] |
|  | Acoustic recorder | 2.84 | 0.87 | 2.65 | 0.72 | 0.86 | 0.393 | [2.56 ,3.12] |
|  | Visual transect | 2.35 | 0.98 | 2.19 | 0.95 | 1.07 | 0.290 | [2.03 ,2.67] |
|  | Forest cover satellite image | 3.28 | 1.02 | 3.56 | 0.97 | -1.24 | 0.222 | [2.94 ,3.61] |
| Time efficient | Camera trap | 1.9 | 1.03 | 1.78 | 0.82 | 0.55 | 0.585 | [1.56 ,2.23] |
|  | Acoustic recorder | 2.85 | 0.76 | 2.73 | 0.77 | 0.57 | 0.570 | [2.60 ,3.09] |
|  | Visual transect | 2.25 | 1.12 | 2.07 | 1.00 | 0.97 | 0.338 | [1.88 ,2.61] |
|  | Forest cover satellite image | 3 | 1.17 | 3.41 | 1.09 | -1.71 | 0.095 | [2.62 ,3.37] |
| Entertaining | Camera trap | 1.89 | 0.85 | 1.87 | 0.74 | 0 | 1 | [1.62 ,2.17] |
|  | Acoustic recorder | 2.87 | 0.76 | 2.85 | 0.72 | 0 | 1 | [2.62 ,3.12] |
|  | Visual transect | 1.74 | 1.01 | 1.80 | 1.03 | -0.24 | 0.814 | [1.41 ,2.07] |
|  | Forest cover satellite image | 3.48 | 0.82 | 3.46 | 1.00 | 0.3 | 0.762 | [3.22 ,3.75] |
| Useful for plants | Camera trap | 2.22 | 0.76 | 2.29 | 0.71 | -0.46 | 0.645 | [1.97 ,2.47] |
|  | Acoustic recorder | 3.65 | 0.53 | 3.85 | 0.42 | -2.08 | 0.043 | [3.47 ,3.82] |
|  | Visual transect | 1.47 | 0.75 | 1.34 | 0.52 | 0.79 | 0.430 | [1.23 ,1.71] |
|  | Forest cover satellite image | 2.65 | 1.07 | 2.51 | 0.92 | 0.68 | 0.498 | [2.30 ,2.99] |
| Useful for mammals | Camera trap | 1.22 | 0.47 | 1.1 | 0.30 | 1.53 | 0.133 | [1.07 ,1.38] |
|  | Acoustic recorder | 3.02 | 0.69 | 2.92 | 0.65 | 0.7 | 0.486 | [2.79 ,3.25] |
|  | Visual transect | 2.15 | 0.69 | 2.2 | 0.68 | -0.33 | 0.743 | [1.92 ,2.38] |
|  | Forest cover satellite image | 3.6 | 0.77 | 3.77 | 0.42 | -1.19 | 0.241 | [3.33 ,3.84] |
| Useful for birds | Camera trap | 2.27 | 0.71 | 2.41 | 0.66 | -0.97 | 0.336 | [2.04 ,2.50] |
|  | Acoustic recorder | 3 | 0.78 | 2.56 | 0.83 | 2.57 | 0.014^b^ | [2.74 ,3.25] |
|  | Visual transect | 1.17 | 0.44 | 1.19 | 0.45 | -0.24 | 0.811 | [1.03 ,1.31] |
|  | Forest cover satellite image | 3.55 | 0.74 | 3.82 | 0.44 | -2.13 | 0.039^b^ | [3.31 ,3.78] |
| Useful for forest status | Camera trap | 2.4 | 0.77 | 2.67 | 0.69 | -2.31 | 0.026^b^ | [2.13 ,2.63] |
|  | Acoustic recorder | 3.65 | 0.66 | 3.62 | 0.66 | 0 | 1 | [3.42 ,3.85] |
|  | Visual transect | 2.22 | 0.76 | 2.37 | 0.86 | -0.45 | 0.653 | [2.01 ,2.49] |
|  | Forest cover satellite image | 1.72 | 1.19 | 1.32 | 0.82 | 2.57 | 0.014^b^ | [1.32 ,2.11] |
| Safe from intentional damage | Camera trap | 3.66 | 0.92 | 3.28 | 1.16 | 1.2 | 0.238 | [3.31 ,3.96] |
|  | Acoustic recorder | 2.64 | 0.70 | 2.76 | 0.63 | -0.84 | 0.405 | [2.39 ,2.88] |
|  | Visual transect | 2.23 | 0.70 | 2.39 | 0.71 | -0.97 | 0.337 | [1.97 ,2.46] |
|  | Forest cover satellite image | 1.46 | 0.82 | 1.55 | 1.10 | 0 | 1 | [1.21 ,1.78] |
| Safe from natural risk | Camera trap | 2.5 | 1.22 | 3.21 | 1.03 | -3.12 | 0.003^c^ | [2.09 ,2.90] |
|  | Acoustic recorder | 3.05 | 0.80 | 3.02 | 0.68 | 0.16 | 0.871 | [2.78 ,3.31] |
|  | Visual transect | 2.39 | 0.88 | 2.51 | 0.86 | -0.88 | 0.383 | [2.10 ,2.68] |
|  | Forest cover satellite image | 2.05 | 1.29 | 1.24 | 0.62 | 3.48 | 0.001^c^ | [1.62 ,2.47] |
| ^a^Ranking responses in each criterion for each the four tools from 1 to 4 where 1 indicates best ranked and 4 the worst  ranked.  ^b^Significance at 5%.  ^c^Significance at 1%. | | | | | | | | |

**Appendix S4.** Survey questionnaires.

**Baseline survey**

0.1. Nombre encuestador/a

0.2. Fecha

1.1. Ejido

1.2. Nombre completo

1.3. Codigo de participante

1.4. Edad

1.5. Sexo

1.6. Escolaridad: ultimo grado escolar

1.7. ¿Cuantas personas viven en su casa, incluyendo usted?

1.8. ¿Cuantas personas de su hogar generan ingresos, incluyendo usted?

1.9. ¿Cual es el numero total de hectareas de sus TIERRAS INDIVIDUALES al dia de hoy?

1.10. ¿Cual es el numero total hectareas de sus TIERRAS DE USO COMUN al dia de hoy?

1.11. ¿Es usted ejidatario/a actualmente?

1.12. Si usted es ejidatario/a, ¿desde cuando?

1.13. ¿Indique cual de las siguientes opciones aplica para usted?

1.14. ¿Cual es su principal ocupación? Indique solamente una

1.15. Si contesto "otro" en la pregunta anterior, indique su actual ocupacion:

1.16. ¿Cuál es su principal fuente de ingresos? Indique solamente una opcion

1.17. Si contesto "otro" en la pregunta anterior, indique su principal fuente de ingresos:

1.18. ¿A cuanto ascendieron los ingresos de su hogar el ultimo mes? (poner numero completo, ej. 10000)

1.19. ¿A cuanto ascienden los ingresos de su hogar en un mes normal?

1.20. ¿A cuanto ascendieron los ingresos de su hogar en el ultimo año?

1.21. ¿Cuanto dinero suele recibir anualmente de PSA en su familia?

1.22. ¿Cuanto dinero recibio el año pasado de PSA?

1.23. ¿Que porcentaje de sus ingresos anuales en el ultimo año vino de PSA?

1.24. ¿Que porcentaje de sus tierras (incluyendo parcelas individuales y tierras de uso comun) estan inscritas actualmente en PSA?

Razones para participar en este estudio. Para cada una de las siguientes razones indique si usted esta: 1= muy en desacuerdo, 2= poco desacuerdo, 3= poco de acuerdo, 4= muy de acuerdo, -8 = no lo sabe

2.1. Espero recibir algun beneficio economico

2.2. Me interesa aprender sobre la biodiversidad en mi comunidad

2.3. Me interesa aprender sobre metodos de monitoreo de biodiversidad

2.4. Solo participo porque es un compromiso de CONAFOR para participantes de PSA

2.5. Participo sobretodo por que otras personas de mi comunidad asistieron

2.6. Me interesa apoyar al trabajo del asesor tecnico de PSA Natura Mexicana

2.7. Me importa mejorar la conservacion de la biodiversidad en mi comunidad

2.8 ¿Por que cree usted que otras personas en su comunidad decidieron NO asistir a este taller sobre monitoreo de biodiversidad

Para las siguientes medidas del estado de conservacion y servicios ambientales en su comunidad, indique si en ultimos 3-4 años usted cree que: 1 = ha disminuido, 2 = ha permanecido igual, 3 = ha aumentado, -8 = no lo sabe

3.1. Cantidad de bosque

3.2. Cantidad de especies de animales

3.3. Cantidad de especies de árboles

3.4. Calidad del suelo

3.5. Calidad del aire

3.6. Cantidad de lluvia

3.7. Cantidad de agua en rios y riachuelos

3.8. Cantidad de incendios

3.9. Temperatura

3.10. Inundaciones

3.11. ¿Nota algunas diferencias entre el estado de conservacion y de servicios ambientales en los sitios inscritos en PSA y los sitios fuera de PSA? ¿Cuales?

¿Ha utilizado alguna de las siguientes herramientas para monitorear la biodiversidad?

4.1. Coleccion de especimenes

4.2. Transectos o recorridos visuales con binoculares

4.3. Imagenes espaciales para analizar deforestacion

4.4. Imagenes espaciales de medicion de luz

4.5. Camaras trampa

4.6. Drones

4.7. Microfonos

4.8. Analisis de material genetico (ADN) ambiental

¿Que tanto le interesa monitorear lo siguiente? 1= NO para nada, 2= NO poco, 3= SI poco, 4= SI mucho, -8 No lo sabe

4.9. Cobertura forestal o deforestacion

4.10. Mamiferos

4.11. Aves

4.12. Reptiles

4.13. Anfibios

4.14. Peces

4.15. Insectos

4.16. Plantas

4.17. Hongos

¿Que tanto le interesa utilizar los siguientes métodos de monitoreo? 1= NO para nada, 2= NO poco, 3= SI poco, 4= SI mucho, -8 No lo sabe

4.18. Coleccion de especimenes

4.19. Transectos o recorridos visuales con binoculares

4.20. Imagenes espaciales para analizar deforestacion

4.21. Imagenes espaciales de medicion de luz

4.22. Camaras trampa

4.23. Drones

4.24. Microfonos

4.25. Analisis de material genetico (ADN) ambiental

4.25. Para la flora y fauna que mas le interese monitorear, indique que especies especificas le gustaria monitorear:

4.26. Ordene las siguientes herramientas de acuerdo lo siguiente: Es facil aprender como funciona. Donde 1 es lo mas FACIL y 4 es lo mas DIFICIL.

Camaras trampa

Microfonos

Transectos visuales con binoculares

Imagenes espaciales para analizar deforestacion

4.27. Ordene las siguientes herramientas de acuerdo lo siguiente: Es facil usarlo y recolectar datos. Donde 1 es lo mas FACIL y 4 es lo mas DIFICIL.

Camaras trampa

Microfonos

Transectos visuales con binoculares

Imagenes espaciales para analizar deforestacion

4.28. Ordene las siguientes herramientas de acuerdo lo siguiente: Toma poco tiempo recolectar datos. Donde 1 es lo que toma MENOS tiempo y 4 es lo que toma MAS tiempo.

Camaras trampa

Microfonos

Transectos visuales con binoculares

Imagenes espaciales para analizar deforestacion

4.29. Ordene las siguientes herramientas de acuerdo lo siguiente: Es divertido o entretenido usar la herramienta. Donde 1 es lo MAS divertido/entretenido y 4 es lo MENOS divertido/entretenido.

Camaras trampa

Microfonos

Transectos visuales con binoculares

Imagenes espaciales para analizar deforestacion

4.30. Ordene las siguientes herramientas de acuerdo lo siguiente: Es util para identificar plantas. Donde 1 es lo mas UTIL y 4 es lo mas INUTIL.

Camaras trampa

Microfonos

Transectos visuales con binoculares

Imagenes espaciales para analizar deforestacion

4.31. Ordene las siguientes herramientas de acuerdo lo siguiente: Es util para identificar mamiferos. Donde 1 es lo mas UTIL y 4 es lo mas INUTIL.

Camaras trampa

Microfonos

Transectos visuales con binoculares

Imagenes espaciales para analizar deforestacion

4.32. Ordene las siguientes herramientas de acuerdo lo siguiente: Es util para identificar aves. Donde 1 es lo mas UTIL y 4 es lo mas INUTIL.

Camaras trampa

Microfonos

Transectos visuales con binoculares

Imagenes espaciales para analizar deforestacion

4.33. Ordene las siguientes herramientas de acuerdo lo siguiente: Es util para ver el estado de conservacion del bosque. Donde 1 es lo mas UTIL y 4 es lo mas INUTIL. Camaras trampa Microfonos Transectos visuales con binoculares Imagenes espaciales

Camaras trampa

Microfonos

Transectos visuales con binoculares

Imagenes espaciales para analizar deforestacion

4.34. Ordene las siguientes herramientas de acuerdo lo siguiente: Es dificil que alguien robe o dañe el equipo intencionalmente. Donde 1 es lo mas DIFICIL y 4 es lo mas FACIL.

Camaras trampa

Microfonos

Transectos visuales con binoculares

Imagenes espaciales para analizar deforestacion

4.35. Ordene las siguientes herramientas de acuerdo lo siguiente: Es dificil que el equipo se dañe por condiciones naturales (ej. humedad, sol, o lluvias). Donde 1 es lo mas DIFICIL y 4 es lo mas FACIL.

Camaras trampa

Microfonos

Transectos visuales con binoculares

Imagenes espaciales para analizar deforestacion

**Endline survey**

6.1. Nombre encuestador/a

6.2. Fecha

7.1. Ejido

7.2. Nombre completo

7.3. Codigo de participante

¿Que tanto le interesa monitorear lo siguiente? 1= NO para nada, 2= NO poco, 3= SI poco, 4= SI mucho, -8 No lo sabe

8.1. Cobertura forestal o deforestacion

8.2. Mamiferos

8.3. Aves

8.4. Reptiles

8.5. Anfibios

8.6. Peces

8.7. Insectos

8.8. Plantas

8.9. Hongos

¿Que tanto le interesa utilizar los siguientes metodos de monitoreo? 1= NO para nada, 2= NO poco, 3= SI poco, 4= SI mucho, -8 No lo sabe

8.10. Coleccion de especimenes

8.11. Transectos o recorridos visuales con binoculares

8.12. Imagenes espaciales para analizar deforestacion

8.13. Imagenes espaciales de medicion de luz

8.14. Camaras trampa

8.15. Drones

8.16. Microfonos

8.17. Analisis de material genetico (ADN) ambiental

8.18. Cuando estuvo en campo realizando transectos visuales para para monitorear biodiversidad, ¿paso algo que sorprendio o llamo la atencion ¿Que fue y por que le sorprendio o llamo la atencion?

8.19. Cuando le presentaron resultados y datos de transectos visuales para monitorear la biodiversidad, ¿hubo algo que sorprendio o llamo la atencion? ¿Que fue y por que le sorprendio o llamo la atencion?

8.20. Cuando estuvo en campo utilizando camaras trampa para monitorear biodiversidad, ¿paso algo que le sorprendio o llamo la atencion? ¿Que fue y por que le sorprendio o llamo la atencion?

8.21. Cuando le presentaron resultados y datos de camaras trampa para monitorear biodiversidad, ¿hubo algo que le sorprendio o llamo la atencion? ¿Que fue y por que le sorprendio o llamo la atencion?

8.22. Cuando estuvo en campo utilizando microfonos para monitorear biodiversidad, ¿paso algo que sorprendio o llamo la atencion?¿Que fue y por que le sorprendio o llamo la atencion?

8.23. Cuando le presentaron resultados y datos de microfonos para monitorear biodiversidad, ¿hubo algo que le sorprendio o llamo la atencion? ¿Que fue y por que le sorprendio o llamo la atencion?

8.24. Cuando le presentaron resultados de analisis de deforestacion usando imagenes espaciales, ¿hubo algo que sorprendio o llamo la atencion? ¿Que fue y por que le sorprendio o llamo la atencion?

8.25. Ordene las siguientes herramientas de acuerdo lo siguiente: Es facil aprender como funciona. Donde 1 es lo mas FACIL y 4 es lo mas DIFICIL.

Camaras trampa

Microfonos

Transectos visuales con binoculares

Imagenes espaciales para analizar deforestacion

8.26. Ordene las siguientes herramientas de acuerdo lo siguiente: Es facil usarlo y recolectar datos. Donde 1 es lo mas FACIL y 4 es lo mas DIFICIL.

Camaras trampa

Microfonos

Transectos visuales con binoculares

Imagenes espaciales para analizar deforestacion

8.27. Ordene las siguientes herramientas de acuerdo lo siguiente: Toma poco tiempo recolectar datos. Donde 1 es lo que toma MENOS tiempo y 4 es lo que toma MAS tiempo.

Camaras trampa

Microfonos

Transectos visuales con binoculares

Imagenes espaciales para analizar deforestacion

8.28. Ordene las siguientes herramientas de acuerdo lo siguiente: Es divertido o entretenido usar la herramienta. Donde 1 es lo MAS divertido/entretenido y 4 es lo MENOS divertido/entretenido.

Camaras trampa

Microfonos

Transectos visuales con binoculares

Imagenes espaciales para analizar deforestacion

8.29. Ordene las siguientes herramientas de acuerdo lo siguiente: Es util para identificar plantas. Donde 1 es lo mas UTIL y 4 es lo mas INUTIL.

Camaras trampa

Microfonos

Transectos visuales con binoculares

Imagenes espaciales para analizar deforestacion

8.30. Ordene las siguientes herramientas de acuerdo lo siguiente: Es util para identificar mamiferos. Donde 1 es lo mas UTIL y 4 es lo mas INUTIL.

Camaras trampa

Microfonos

Transectos visuales con binoculares

Imagenes espaciales para analizar deforestacion

8.31. Ordene las siguientes herramientas de acuerdo lo siguiente: Es util para identificar aves. Donde 1 es lo mas UTIL y 4 es lo mas INUTIL.

Camaras trampa

Microfonos

Transectos visuales con binoculares

Imagenes espaciales

8.32. Ordene las siguientes herramientas de acuerdo lo siguiente: Es util para ver el estado de conservacion del bosque. Donde 1 es lo mas UTIL y 4 es lo mas INUTIL. Camaras trampa Microfonos Transectos visuales con binoculares Imagenes espaciales

Camaras trampa

Microfonos

Transectos visuales con binoculares

Imagenes espaciales para analizar deforestacion

8.33. Ordene las siguientes herramientas de acuerdo lo siguiente: Es dificil que alguien robe o dañe el equipo intencionalmente. Donde 1 es lo mas DIFICIL y 4 es lo mas FACIL.

Camaras trampa

Microfonos

Transectos visuales con binoculares

Imagenes espaciales para analizar deforestacion

8.34. Ordene las siguientes herramientas de acuerdo lo siguiente: Es dificil que el equipo se dañe por condiciones naturales (ej. humedad, sol, o lluvias). Donde 1 es lo mas DIFICIL y 4 es lo mas FACIL.

Camaras trampa

Microfonos

Transectos visuales con binoculares

Imagenes espaciales para analizar deforestacion

Indique que tan de acuerdo esta con las siguientes frases. Monitorear la biodiversidad y comunicar los resultados en mi comunidad… (1= No de acuerdo para nada, 2= No de acuerdo pero poco, 3= Si de acuerdo poco, 4= SI de acuerdo mucho, -8 = no lo sabe)

9.1. Nos ayudaria a conocer mejor el estado de conservacion de los bosques de la comunidad

9.2. Nos ayudaria a conocer mejor la las plantas, mamiferos y aves dentro de los bosques de la comunidad

9.3. Nos ayudaria a identificar sitios donde puede haber conflicto entre animales silvestres y animales domesticos y/o cultivoa (ej. felinos y ganado, o tejones y cultivo de maiz)

9.4. Nos ayudaria a reducir la tala ilegal

9.5. Nos ayudaria a reducir la caza ilegal

9.6. Nos ayudaria a reducir la pesca ilegal

9.7. Nos ayudaria a generar mas conciencia para la conservación

9.8. Nos ayudaria a mejorar el control y prevencion de incendios

9.9. Nos ayudaria a conseguir mas financiamiento para PSA

9.10. Podria generar conflictos dentro de la comunidad

Indique que tan de acuerdo esta con las siguientes frases. En comparacion con las personas que no son de nuestra comunidad, participantes de PSA como yo... (1= No de acuerdo para nada, 2= No de acuerdo pero poco, 3= Si de acuerdo poco, 4= SI de acuerdo mucho, -8 = no lo se)(

9.11. Conocemos mejor las condiciones de nuestros bosques

9.12. Sabemos mejor los sitios en donde se pueden encontrar ciertos animales o plantas

9.13. Sabemos mejor en que momentos del dia se pueden monitorear ciertos animales o plantas

9.14. Sabemos mejor donde hay actividades ilicitas

9.15. Nos podemos organizar mejor para planear y realizar actividades

9.16. Nos podriamos poner en situacion de riesgo si documentamos actividades ilegales como caceria o tala ilegal

9.17 ¿Quienes cree usted que deberian de PARTICIPAR EN REUNIONES PARA PLANEAR actividades de monitoreo de la biodiversidad en el programa PSA? (marque las que considere(

9.18. ¿Quienes cree usted que deberian de REALIZAR LA COLECCION DE DATOS de monitoreo de la biodiversidad en el programa PSA?

9.19. ¿Quienes cree usted que deberian de REALIZAR EL ANALISIS DE DATOS de monitoreo de la biodiversidad en el programa PSA?

9.20. ¿Quienes cree usted que deberian de PARTICIPAR EN ACTIVIDADES DE COMUNICACION DENTRO DE SU COMUNIDAD de resultados de monitoreo de la

biodiversidad en el programa PSA?

9.21. ¿Quienes cree usted que deberian de PARTICIPAR EN ACTIVIDADES DE COMUNICACION FUERA DE SU COMUNIDAD de resultados de monitoreo de la biodiversidad en el programa PSA?

9.22. ¿Quienes cree usted que deberian de FINANCIAR REUNIONES PARA PLANEAR de monitoreo de la biodiversidad en el programa PSA?

9.23. ¿Quienes cree usted que deberian de FINANCIAR LA COLECCION DE DATOS de monitoreo de la biodiversidad en el programa PSA?

9.24. ¿Quienes cree usted que deberian de FINANCIAR EL ANALISIS DE DATOS de monitoreo de la biodiversidad en el programa PSA?

9.25. ¿Quienes cree usted que deberian de FINANCIAR ACTIVIDADES DE COMUNICACION DENTRO DE SU COMUNIDAD de resultados de monitoreo de la biodiversidad en el programa PSA?

9.26. ¿Quienes cree usted que deberian de FINANCIAR ACTIVIDADES DE COMUNICACION FUERA DE SU COMUNIDAD de resultados de monitoreo de la biodiversidad en el programa PSA?

9.27 ¿Que porcentaje de ejidatario/as en su ejido son mujeres?

9.28 ¿Que porcentaje de ejidatario/as que participan en PSA en su ejido son mujeres?

9.29. ¿Usted cree que deberian de participar mas mujeres en el monitoreo de la biodiversidad en PSA?

9.30. Explique su respuesta a la pregunta anterior. ¿En qué actividades podrían o no participar las mujeres?

9.31. ¿Como cree usted que seria util comunicar los resultados del monitoreo de la biodiversidad en PSA dentro de su comunidad?

Indique que tanto le interesaria participar en las siguientes actividades asociadas a monitoreo de la biodiversidad durante la duracion de su contrato de PSA: 1= NO para nada, 2= NO poco, 3= SI poco, 5= SI mucho, -8= no lo sabe

10.1. Reuniones con personal de CONAFOR de PSA para planear actividades de monitoreo

10.2. Reuniones con personal de asesores tecnicos de PSA como Natura Mexicana para planear actividades de monitoreo

10.3. Reuniones con miembros de la comunidad para planear actividades de monitoreo

10.4. Coleccion de datos en campo

10.5. Analisis y manejo de datos de monitoreo

10.6. Comunicacion de resultados de datos de monitoreo con miembros de su comunidad

10.7. Comunicacion de resultados de datos de monitoreo con el gobierno

10.8. Comunicacion de resultados de datos de monitoreo con otras comunidades en su region

Indique que tanto le podrian impedir los siguientes factores el poder involucrarse en monitoreo de la biodiversidad durante la duracion de su contrato de PSA. 1= NO para nada, 2= NO poco, 3= SI poco, 4= SI mucho, -8= no lo sabe

10.9. Falta de tiempo

10.10. Falta de apoyo economico

10.11. Falta de interes

10.12. Se puede volver aburrido o cansado

10.13. Falta de conocimiento tecnico

10.14. Puede ser peligroso
